# The Natural Effect of BCG Vaccination on COVID-19: The Debate Continues

**DOI:** 10.3389/fimmu.2022.953228

**Published:** 2022-07-08

**Authors:** Wenping Gong, Huiru An, Jie Wang, Peng Cheng, Yong Qi

**Affiliations:** ^1^ Tuberculosis Prevention and Control Key Laboratory, Senior Department of Tuberculosis, The 8^th^ Medical Center of People's Liberation Army (PLA) General Hospital, Beijing, China; ^2^ Beijing Key Laboratory of New Techniques of Tuberculosis Diagnosis and Treatment, Senior Department of Tuberculosis, The 8th Medical Center of People’s Liberation Army (PLA) General Hospital, Beijing, China; ^3^ Huadong Research Institute for Medicine and Biotechniques, Nanjing, China

**Keywords:** BCG (Bacille Calmette-Guérin), COVID-19, *Mycobacterium tuberculosis*, clinical trial, hospitalization, protective effect

## Introduction

The coronavirus disease 2019 (COVID-19) pandemic caused by severe acute respiratory syndrome coronavirus 2 (SARS-CoV-2) infection has inflicted an unprecedented and significant toll on global public health and the economy. During the fight against the pandemic, the century-old tuberculosis (TB) vaccine Bacille Calmette Guérin (BCG), which has been reported to protect against infections of various respiratory pathogens other than *Mycobacterium tuberculosis* by inducing non-specific immune responses, was found to play an essential role against COVID-19 in various ecological, analytical, and animal studies (1, 2). However, its effectiveness remains controversial due to the limited number of published clinical trials (2). The effect of BCG vaccines appears to be quite non-specific or speculative. Currently there is no clear or strong evidence to support the notion that BCG vaccination confer protection to COVID-19. In the absence of evidence, the World Health Organization (WHO) issued a Scientific Brief on April 12, 2020, against BCG vaccination to prevent COVID-19, and this recommendation has remained unchanged to date (3).

## Non-Specific Immune Response Induced by the BCG

Our previous studies have reported that BCG vaccination could induce a non-specific immune response to fight against unrelated pathogens (2, 4–7). The non-specific immune response induced by BCG vaccination may be due to three potential mechanisms: trained immunity, heterologous immunity, and anti-inflammatory effect (2). Of the three possible mechanisms, trained immunity has received the most attention. Trained immunity is derived from immune memory in innate immune cells such as monocytes and macrophages. The primary immunization of BCG can activate innate immune cells to produce IL-1β, TNF-α, and IL-6, generating trained monocytes/macrophages with an “infection memory.” These trained monocytes/macrophages will respond rapidly by releasing higher levels of cytokines to combat the second unrelated pathogens’ invasion (8). The trained immunity induced by BCG has been demonstrated in both animal experiments and human clinical trials, and it may be beneficial for the prevention and treatment of SARS-CoV-2 infection (2, 4–7, 9–11).

## Early Evidence on BCG Prevention of COVID-19

Early in the COVID-19 outbreak, the hypothesis that BCG could prevent COVID-19 infection was raised. Isabel N. Kantor was the first to publicly discuss whether BCG has preventive and protective effects against COVID-19 and how strong the association is between the “more BCG vaccination” and the “ less COVID-19 infection” (12). Then, the lead investigator of the BRACE trial (NCT04327206) and the BCG-CORONA trial (NCT04328441) as well as the Director General of WHO discussed this hypothesis, and they suggested that BCG might reduce viremia following SARS-COV-2 exposure, thereby reducing the severity of COVID-19 and recovering faster (13). Furthermore, Luke A. J. O’Neill and Mihai G. Netea claimed that the BCG vaccine might well be a bridge to a specific COVID-19 vaccine due to the trained immunity induced by BCG.

Although this hypothesis offered a glimmer of hope to the panicked and helpless people in the early days of the COVID-19 pandemic, it still needed a lot of evidence to prove it. In the early months of the COVID-19 pandemic, findings from the ecological and analytical studies indicated that countries with low BCG coverage had significantly higher rates of COVID-19 morbidity and mortality than countries with high BCG coverage (14–40). On the contrary, other ecological and analytical studies found that BCG vaccination could not provide adequate protection against COVID-19 infection (41–50). The results of these ecological and analytical studies were contradictory, and the heterogeneity of these findings may originate from some confounding factors, such as population density, ethnicity, age structure, income, healthcare access and quality index, COVID-19 transmission progression, COVID-19 testing rate, non-pharmaceutical interventions, and geographical distribution (2, 5, 51–53). Therefore, these findings should be investigated by randomized clinical trials.

## Latest Evidence on BCG Prevention of COVID-19

Recently, results of a double-blind, randomized, placebo-controlled clinical trial (NCT04379336), which aimed to evaluate the efficacy of BCG vaccination in delivering protection against COVID-19 in healthcare workers (HCWs) in South Africa, were published in the journal *eClinicalMedicine* and attracted wide attention (54). The study reported that BCG revaccination failed to protect HCWs from SARS-CoV-2 infection, severe COVID-19, hospitalization, and respiratory tract infections (RTIs), on the contrary, resulting in an unexpected trend toward more symptomatic and severe RTIs. Nevertheless, it is plausible that BCG offered protection from death. The results indicated that BCG had no statistically significant effect on COVID-19, which in our opinion, maybe still controversial.

A large number of COVID-19 immune-tolerant rather than sensitive participants recruited in the study may have influenced or masked the true protective effect of the BCG vaccine. However, the COVID-19-seropositive population, with a rate of 15.3% resistant to COVID-19-related severe disease, were not excluded. Considering that *M. tuberculosis*-infected mice were resistant to secondary SARS-CoV-2 infection (48), it’s not inconceivable that the latent tuberculosis infection in the HCWs occupied 48.5% of all the participants might play resistant roles and influence the significant difference between groups. However, they were not excluded, either. More importantly, all the participants had previously received at least one dose of the BCG vaccine due to the vaccination policy of South Africa before the trial, which also could decrease the variation between the BCG revaccination and placebo groups. Interestingly, the only clinical trial to obtain results similar to this one also recruited volunteers who had received the BCG vaccine before in Poland (55), indicating the limitation should not be ignored.

Considering that many clinical trials began in the early days of the pandemic like this study, there must have been various drawbacks in their design due to the lack of understanding of the disease and its interaction with the BCG vaccine at that time. For instance, the BCG vaccination-caused scars would expose the participants’ group and make the double-blind experimental design impossible. Furthermore, the young and middle-aged participants being relatively more resistant to COVID-19 than older people may mask the real efficacy of the BCG vaccine. Here we summarize the designs, results, and deficiencies of all published clinical studies (**Table 1**) (55–59) and hope to provide helpful information for more clinical studies involving the efficacy of BCG vaccination against COVID-19 or other virus infections in the future. After all, though more than 50 clinical trials have been registered, results of less than ten clinical trials have been published so far (2).

**Table 1:** Summary of the designs, results, and deficiencies of all published clinical studies.

	NCT04379336 (54)	NCT04414267 (56)	NCT03296423 (57)	NCT04648800 (55)	NCT04475302 (58)	NCT04537663 (59)
Sample size	1. 1000 healthcare workers were recruited from private and public healthcare facilities 2. 126 participants assigned to receive placebo; 139 participants assigned to receive BCG through the trail.	1. Elderly Greek individuals were randomized (1:1) to BCG revaccination or placebo group 2. Baseline characteristics are comparable between the two arms of vaccination 516 eligible participants 3. 6-month analysis was performed in 98 placebo-vaccinated individuals and 92 BCG-vaccinated individuals.	1. 202 elderly Greek individuals were enrolled; 4 participants withdrew consent. 2. Interim analysis included 78 participants in placebo group and 72 participants in BCG group	1. 717 HCWs were recruited, 363 (50.6%) individuals were excluded for Tuberculin test positive results. 2. 177 participants were randomly assigned to BCG group, and 177 to placebo group.	86 participants, Experimental group: 54 participants received 0.1 ml BCG intradermal injection. Control group: 32 participants were not vaccinated	6132 participants。 Participants will be randomized between intracutaneous administration of BCG vaccine (Danish strain 1331) or placebo (0.1ml 0.9% NaCl) in a 1:1 ratio.
Age (years)	39-40	>50	>65	>25	60-80	≥60
BCG strain	Danish strain 1331	Moscow strain 361- I	Danish strain 1331	Brazilian strain	NA	Danish strain 1331
Inclusion criteria	1. 15.3% of participants had a positive serology test indicating prior SARSCoV-2 exposure with no known history of COVID-19. 2. 485(48.5%) of participants had a positive QuantiFERON Gold Plus result, indicating have latent TB infection 3. adult healthcare workers, defined as any personnel working in a healthcare facility expected to be highly exposed to COVID-19,	Enrolled people should also have skin tuberculin test diameter less than 10mm and negative serum testing for immunoglobulin G and M against SARS-CoV-2.	The study enrolled elderly patients (age ≥ 65 years) of both genders who were discharged from hospital after hospitalization for a medical cause.	1. A health care professional (physician, nurse, midwife, paramedic, electroradiology technician, laboratory diagnostician, physiotherapist, nutritionist, orderly) aged > 25 years 2. No confirmed SARS-CoV-2 infection 3. Earlier vaccination against tuberculosis 4. Receive two doses of the COVID-19 vaccine as part of the National Immunization Program after December 27, 2020	“1. Elderly individuals between 60 - 80 years of age residing in hotspots for SARS-Cov2 infection were included in the study between July 2020 and September 2020 in Chennai, India. 2. No known history of HIV or on immunosuppressive drugs for malignancy or transplant”	“1. Age ≥60 years 2. Having a chronic disease or having undergone major surgery 3. Meeting at least one of the following criteria: (1).Planned to be discharged from the hospital or discharged from the hospital less than 6 weeks ago. Departments of interest are those that in the opinion of the principle investigator admit mostly vulnerable elderly and include but are not limited to: cardiology, pulmonology, internal medicine, neurology. (2).Visiting a medical outpatient clinic (3).Attending the thrombosis care service”
Exclusion criteria	1. Respiratory tract or other active infection, 2. COVID-19 treatment, 3. Contraindication to the BCG vaccine including known hypersensitivity to BCG, pregnancy or were breastfeeding, compromised immune system including HIV and cancer, 4. Receiving immunosuppressive therapy. 5. Previous COVID-19	1. Infection by HIV-1 2. Primary immunodeficiency. 3. Solid organ transplantation 4. Bone marrow transplantation 5. Intake of chemotherapy the last two months 6. Intake of radiotherapy the last two months 7. Active hemalogical or solid tumor malignancy 8. Intake of any anti-cytokine therapies 9. Intake of oral or intravenous steroids defined as daily doses of 10mg prednisone or equivalent for longer than the last 3 months.	1. Denial for written informed consent. 2. Solid organ malignancy or lymphoma diagnosed the last five years. 3. Treatment with oral or intravenous steroids. 4. Severe immunodeficiency, neutropenia, history of solid organ and bone marrow transplantation, intake of chemotherapy, primary immunodeficiency, severe lymphopenia and treatment with anti-cytokine therapies. 5. Positive IFN-γ Release Assay (IGRA)	1. Hypersensitivity to any component of BCG 2. Hypersensitivity to previously administered tuberculin 3. HIV infection 4. Primary or secondary immunodeficiencies 5. Radiotherapy 6. Treatment with corticosteroids, ongoing immunosuppressive therapy 7. Neoplastic diseases 8. After stem cell transplantation and organ transplantation 9. In the exacerbation stage of chronic diseases 10. Pregnancy 11. History of tuberculosis 12. Keloid at the vaccination site after previous BCG vaccination	1. Positive for SARS-Cov2 infection by either antibody (serology) or PCR test 2. HIV-infected or individuals with malignancy or on immunosuppressive drugs or transplant recipients and those on dialysis or anti-psychiatric medications or hypersensitivity to vaccinations	1. Fever (>38°C) within the past 24 hours 2. Suspicion of active viral or bacterial infection 3. Vaccination with live vaccine 4. Infection by HIV-1; neutropenic with less than 500 neutrophils/mm3; solid organ transplantation; bone marrow transplantation; hematological malignancy; chemo-, radio- or immunotherapy for solid organ malignancy; primary immunodeficiency; severe lymphopenia; treatment with any immunosuppressant drugs 5. Known history of a positive Mantoux or active TB 6. Born in a country with high incidence of TB 7. Active participation in another research study 8. History of COVID-19 9. Not able to perform the study procedures 10. Legally incapacitated or unwilling to provide informed consent
Intervention	BCG:0.1mL, placebo:0.1mL NaCl	Subdermal injection of 0.1ml of sodium chloride 0.9% or with 0.1ml of BCG vaccine	Intradermal vaccination with 0.1ml of sodium chloride 0.9% or with 0.1ml of BCG	Intradermal vaccination with 0.1ml of sodium chloride 0.9% or with 0.1ml of BCG	BCG: Single dose of 0.1ml of BCG vaccine. Placebo: No BCG vaccine	Intradermal injection in the left upper arm with BCG. Placebo: Intradermal injection of sterile 0.9% NaCl
Morbidity	A total of 99 COVID-19 event were record on BCG, and 93 COVID-19 events were record on placebo.	During these first 3 months after the vaccination, the overall incidence of COVID-19 was 10 patients in placebo *vs*. two patients in BCG group, p=0.086. In contrast, 6-months after vaccination, the total number of COVID-19 diagnoses (possible/probable/definitive) was significantly lower in the BCG-vaccinated group compared with the placebo group: OR 0.32 in multivariate analysis (95% CI 0.13-0.79, p=0.014)	The incidence of new infection was 42.3% (95% confidence intervals [CIs] 31.9%–53.4%) in the placebo group and 25.0% (95% CIs 16.4%–36.1%) in the BCG group.	COVID-19 events occurred in 161 participants among the BCG vaccinated people (23.16%) and was absent in 534 (76.84%) of the BCG non-vaccinated group.	NA	NA
Severe disease rate	A higher proportion of a total of 27 severe COVID-19 events were recorded on BCG (19, 70.4%) compared to placebo (8, 29.6%)	Definitive diagnosis of severe COVID-19 requiring hospitalization was present in 5 individuals in the placebo group and only one in the BCG group	NA	A serious adverse event (SAE) with hospitalization for COVID-19 occurred in one female participant	NA	
Hospitalization	1. The primary endpoint of hospitalization due to COVID-19 occurred in 15 (1.5%) participants: 10 (67%) on BCG compared to 5 (33%) on placebo, with an HR of 2.0 (95% CI 0.69−5.9,p = 0.20) 2. The time-to-first hospitalization for all causes included 47 (4.7%) admissions; 27 (57.4%) on BCG and 20 (42.6%) on placebo with an HR of 1.36 (95% CI 0.72−2.49, p = 0.31).	NA	NA	NA	NA	
Mortality	Four participants (0.4%) died, two (50.0%) due to COVID-19 and one each (25.0%) due to bowel perforation and a cerebrovascular accident, all on placebo.	NA	NA	NA	NA	
Limitation	1. The lack of information on BCG vaccination at birth. 2. The lower-than-expected attack rate (19.2%) and hospitalisation rate (7.8%) 3. Participants with a positive serology test were included 4. Unblinded within a few days of enrollment 5. Participants with positive QuantiFERON results were included 6. The number of participants included in final analysis was not clear	1. Small sample size 2. Lack of microbiological testing in all patients with a clinical diagnosis of possible or probable COVID-19. 3. No conclusions can be drawn regarding the effect of BCG vaccination on the severe forms of COVID-19 4. Lack of information regarding the SARS-CoV-2 strains 5. Limited information regarding the impact of BCG on the specific immunological host defense pathways against SARS-CoV-2	1. Small sample size 2 The lack of repeat IGRA after vaccination 3. The absence of serological information on the incidence of various respiratory infections 4. The lack of information on BCG vaccination at birth. 5. The number of individuals participating in the trial is too low	1. The lack of a never-vaccinated control population. 2. Small sample size 3. The stimulation of non-specific effects after the BCG vaccine is associated 4. Lacking complete knowledge about all routes of influence of the BCG vaccine	1. Small sample size 2. This study did not examine the mechanical changes in the immune system.	1. Almost all individuals participating in this study received the BCG vaccine for the first time, as the Netherlands did not adopt a policy of vaccination with BCG in childhood. 2. There is no specific outcome data.

NA, not applicable.

## Conclusion

The hypothesis that BCG has a protective effect against COVID-19 has not been robustly verified. Therefore, the interpretation of any clinical trial results used to confirm this hypothesis should follow the principles of objectivity, science, and prudence. Considering the ongoing pandemic and the possibility of novel variants or other pathogens emerging, the potential effect of BCG on COVID-19 needs to be further confirmed in rigorous randomized clinical trials.

## Author Contributions

Conceptualization: WG, YQ, and HA; Data curation: JW and PC; Formal analysis: YQ; Funding acquisition: WG; Methodology: HA, JW, and PC; Writing - original draft: WG and YQ; Writing - review and editing: HA, YQ, and WG. All authors contributed to the article and approved the submitted version.

## Funding

This study was funded by the Beijing Municipal Science & Technology Commission (Grant No. 19L2065) and the Chinese PLA General Hospital (Grant No. QNC19047) to WG.

## Conflict of Interest

The authors declare that the research was conducted in the absence of any commercial or financial relationships that could be construed as a potential conflict of interest.

## Publisher’s Note

All claims expressed in this article are solely those of the authors and do not necessarily represent those of their affiliated organizations, or those of the publisher, the editors and the reviewers. Any product that may be evaluated in this article, or claim that may be made by its manufacturer, is not guaranteed or endorsed by the publisher.
